# Experiments on the Ultrasonic Bonding Additive Manufacturing of Metallic Glass and Crystalline Metal Composite

**DOI:** 10.3390/ma12182975

**Published:** 2019-09-14

**Authors:** Guiwei Li, Ji Zhao, Jerry Ying Hsi Fuh, Wenzheng Wu, Jili Jiang, Tianqi Wang, Shuai Chang

**Affiliations:** 1 School of Mechanical and Aerospace Engineering, Jilin University, Changchun 130025, Chinajzhao@jlu.edu.cn (J.Z.); jiangjl16@mails.jlu.edu.cn (J.J.); wangtq18@mails.jlu.edu.cn (T.W.); 2 Department of Mechanical Engineering, National University of Singapore, Singapore 117576, Singapore; jerry.fuh@nus.edu.sg (J.Y.H.F.); msecs@nus.edu.sg (S.C.); 3 School of Mechanical Engineering and Automation, Northeastern University, Shenyang 110004, China

**Keywords:** metallic glasses, composite materials, interfaces, additive manufacturing, ultrasonic bonding, 3D printing

## Abstract

Ultrasonic vibrations were applied to weld Ni-based metallic glass ribbons with Al and Cu ribbons to manufacture high-performance metallic glass and crystalline metal composites with accumulating formation characteristics. The effects of ultrasonic vibration energy on the interfaces of the composite samples were studied. The ultrasonic vibrations enabled solid-state bonding of metallic glass and crystalline metals. No intermetallic compound formed at the interfaces, and the metallic glass did not crystallize. The hardness and modulus of the composites were between the respective values of the metallic glass and the crystalline metals. The ultrasonic bonding additive manufacturing can combine the properties of metallic glass and crystalline metals and broaden the application fields of metallic materials.

## 1. Introduction

Metallic glass (MG), also known as amorphous alloy or liquid metal, is produced via modern rapid-solidification metallurgy [1,2]. The internal atoms are arranged in a short-range-ordered, long-range-disordered amorphous structure due to the rapid cooling of liquid melt [3]. It has the excellent mechanical, physical, and chemical properties of metals and glass and has broad application prospects in the automotive, aerospace, medical, communication, and industrial automation fields [4,5,6,7,8,9,10,11,12]. However, these materials have glassy interior microstructures, making them brittle, and their critical forming sizes make them difficult to manufacture bulk blanks, limiting their applications [13]. Recently, some researchers have employed traditional additive manufacturing technologies to manufacture bulk metallic glass, which still cannot improve its mechanical properties [14,15,16,17,18,19,20,21,22]. Cu, Al, and other conventional crystalline metals, in contrast, form crystals because of the ordering of their internal atoms. They have high plasticity because of the crystal slip deformation and twinning deformation under stress, but the mechanical properties, such as strength and hardness, are much lower than those of glassy metals [23,24]. To synthesize the advantages of metallic glass and crystalline metals, they can be combined with additive manufacturing to manufacture bulk composites. This has been explored by various researchers, as described below.

Li et al. made Fe-based metallic glass and crystalline Cu composite parts by selective laser melting [25]. Kim et al. employed electron beam welding to bond Zr-based metallic glass and stainless steel, but the surface of the Zr-based metallic glass crystallized easily [26]. Li et al. explored the joint effect of Zr-based metallic glass and crystalline metal by using laser-foil-printing additive manufacturing, and the results illustrated that Zr-based metallic glass can be welded to Zr 702 alloy [27,28]. Feng et al. investigated the fracture mechanism of Zr-based metallic glass and crystalline Cu composites processed by explosive welding [29]. Wang employed laser impact welding to bond Fe-based metallic glass and crystalline Cu and found the interface hardness to be much higher than that of crystalline Cu [30]. 

The above studies mainly focused on the manufacture of bulk metallic glass composites with a high-energy beam, such as laser beams. These methods were very complex, required high-quality raw materials, and often crystallized the metallic glass. An alternative method that might avoid such issues is ultrasonic bonding additive manufacturing. Ultrasonic additive manufacturing is a hybrid additive manufacturing technique, which combines the capabilities of ultrasonic bonding and CNC milling [31,32]. Based on ultrasonic bonding, this method is simpler, has lower quality requirements for raw materials, increases the sample temperature less, and can bond various kinds of materials [33]. In this study, we used a custom ultrasonic bonding system to bond Ni-based metallic glass ribbons with Al and Cu crystalline ribbons, and we analyzed the interfaces of metallic glass and crystalline metal composites processed by ultrasonic bonding additive manufacturing.

## 2. Material and Methods

**Figure 1:**
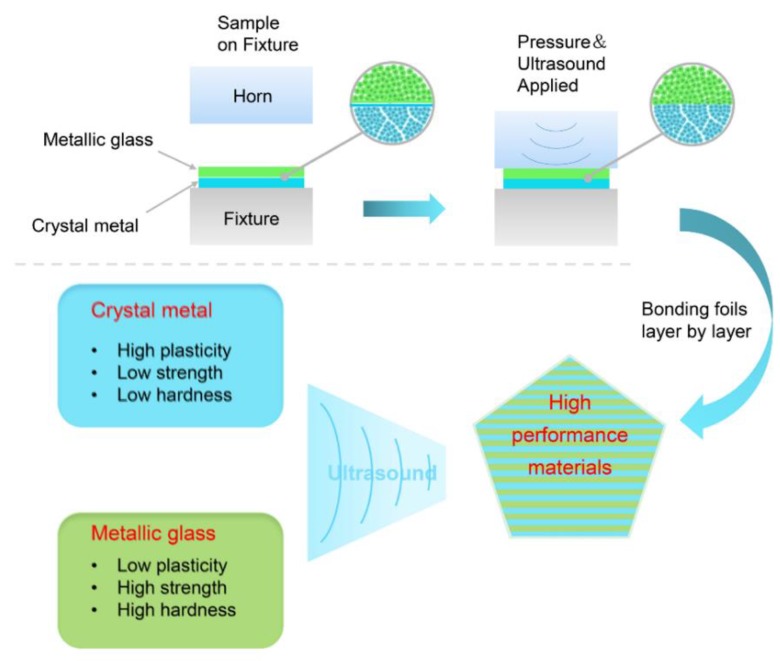
Schematic of ultrasonic bonding additive manufacturing.

Figure 1 shows the principle of ultrasonic bonding additive manufacturing. First, a Ni-based metallic glass ribbon and a crystalline metal ribbon were placed on the fixture, and ultrasonic bonding parameters were set to activate the ultrasonic bonding system. Then, the horn was brought into contact with the ribbon and pressed down to perform ultrasonic consolidation. After ultrasonic consolidation, the specimen remained under pressure from the horn for a short time to prevent the specimen from warping. This consolidation combined the two metal ribbons into one piece, which was then combined with the next metal ribbon until the entire part was completed. This additive manufacturing technology has great potential for manufacturing high-performance bulk metallic glass composites and functional graded materials.

**Figure 2:**
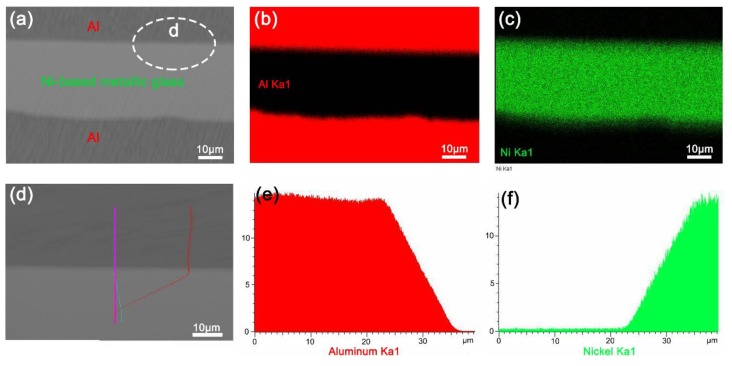
The cross-sectional SEM image of Al/Ni-based (MG) composites (**a**), EDS mapping analysis of Al (**b**) and Ni (**c**) elements, and EDS line analysis of Al (**e**) and Ni (**f**) elements along the pink line of the cross-sectional SEM image (**d**).

Ni_82.2_Cr_7_B_3_Si_4.8_Fe_3_ (wt %) metallic glass ribbons (Miai Metal Material Co. LTD, Kunshan, China), Al ribbons, and Cu ribbons with cross-sectional dimensions of 1.7 × 0.04 mm, 1.7 × 0.1 mm, and 1.7 × 0.1 mm, respectively, were used, considering that Ni-based metallic glass has a good welding capacity [34]. A custom ultrasonic bonding system (Dongguan Jieshi Ultrasonic Automation Co., Ltd., Dongguan, China) was used to bond metallic glass and crystalline metal, using a frequency of 35 kHz and a power of 800 W. Figure 2a shows the cross-sectional morphology of a three-layer Al/Ni-based (MG) composite sample processed by the ultrasonic bonding system. Figure 2b,c show the EDS mapping analysis of the Al and Ni elements in Figure 2a, respectively. Figure 2e,f show the EDS line analysis of the Al and Ni elements along the pink line in Figure 2d, respectively. These two elements diffused slightly at the interfaces. Figure 2 illustrates that metallic glass can be bonded with crystalline metal, and the ultrasonic bonding process can be employed to manufacture bulk metallic glass composites additively with layer-by-layer accumulating formation characteristics. The effects of ultrasonic vibration energy on the quality of the interfaces in the Ni-based metallic glass composites were studied.

During ultrasonic consolidation, the energy inputted via the ultrasonic bonding system into the interior of the consolidated sample is defined as *Q* = *Pt*, where *Q* is the input energy, *P* is the power, and *t* is the ultrasonic bonding time that the ultrasonic wave acted on the ribbons. The time between the ultrasonic emission and the start of the ultrasonic bonding system is the delay time, and the time that the horn continues to press on the ribbons after the ultrasonic emission is the hold time. The bonding time *t* is the main parameter which affects the quality of consolidation between a layer of metallic glass and a layer of crystalline metal. Table 1 shows the experimental scheme.

The cross-sectional morphologies of the consolidated samples were observed by SEM (ZEISS-EVO18, Carl Zeiss NTS, Oberkochen, Germany). The sample cross section was first polished with 8000 mesh sandpaper and then polished on a polisher (Kejing Automation Equipment Co. LTD, Shenyang, China) with a 50 nm SiO_2_ polishing liquid. The bonding interface of the torn sample was observed using a digital microscope (Keyence Singapore PTE LTD, Singapore, Singapore). The phase composition of the bonding interface of the sample was tested by XRD (X’ Pert PRO MPD, PANalytical BV, Almelo, Netherlands) using Cu Kα radiation at λ = 1.54 Å, operated at 40 kV and 40 mA. The interior hardness and modulus of the consolidated samples were tested by nanoindentation (Nano Indenter G200, Agilent, Oak Ridge, USA) using a peak holding time of 3 s and a surface approach velocity of 10 nm/s.

## 3. Results and Discussion

### 3.1. SEM of the Cross-Sectional Morphology

**Figure 3:**
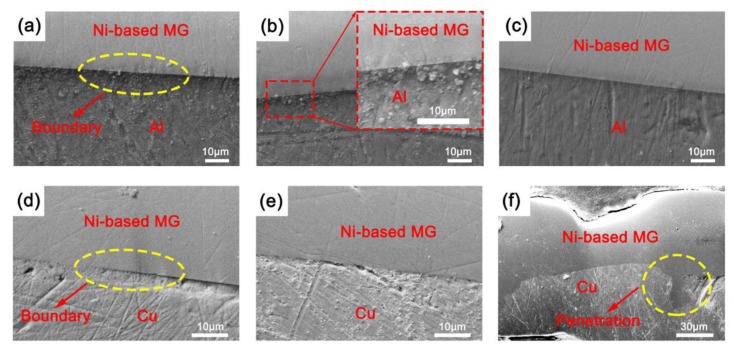
Cross-sectional morphologies of Al/Ni-based (MG) composite samples (**a**–**c**) at bonding times of 60, 80, and 160 ms, as well as cross-sectional morphologies of Cu/Ni-based (MG) composite samples (**d**–**f**) at bonding times of 40, 60, and 140 ms.

Figure 3 shows the cross-sectional morphologies of the metallic glass and crystalline metal composites at various bonding times. After polishing, the surfaces of the metallic glass were relatively smooth, but the surfaces of the crystalline metals showed some obvious scratches. This result occurred mainly because the atomic arrangement inside the metallic glass had short-range order, long-range disorder, and better wear resistance than the crystalline metal. The metallic glass is much harder than Al or Cu, and under the same polishing force, slightly more of the crystalline metal was removed than the Ni-based metallic glass.

Figure 3a–c show a clear boundary between the Ni-based metallic glass and Al but no gaps at the interfaces. The consolidation interfaces could remain relatively flat with the increase of bonding times, indicating that the metallic glass and Al can be bonded well over a wide range of bonding times. Figure 3d,e show that the bonding interfaces between the Ni-based metallic glass and Cu are relatively flush and there are no gaps at the interfaces. However, when the bonding time was 140 ms, the joint interface appeared corrugated, and some areas showed penetration of the Ni-based metallic glass into the Cu. Thus, Ni-based metallic glass can be bonded well to Cu only over a limited range of ultrasonic vibration energies. This may be connected with the ductility of the crystalline metals. Al is more malleable than Cu and tends to form a stable plastic flow when forming a welding joint. Since the metallic glass is harder than the Cu, the excessive input energy from the ultrasonic bonding system into the bonding sample may have resulted in an unstable plastic flow of raw materials, which made the metallic glass penetrate into Cu. 

### 3.2. Phase Analysis at the Joint Interface

**Figure 4:**
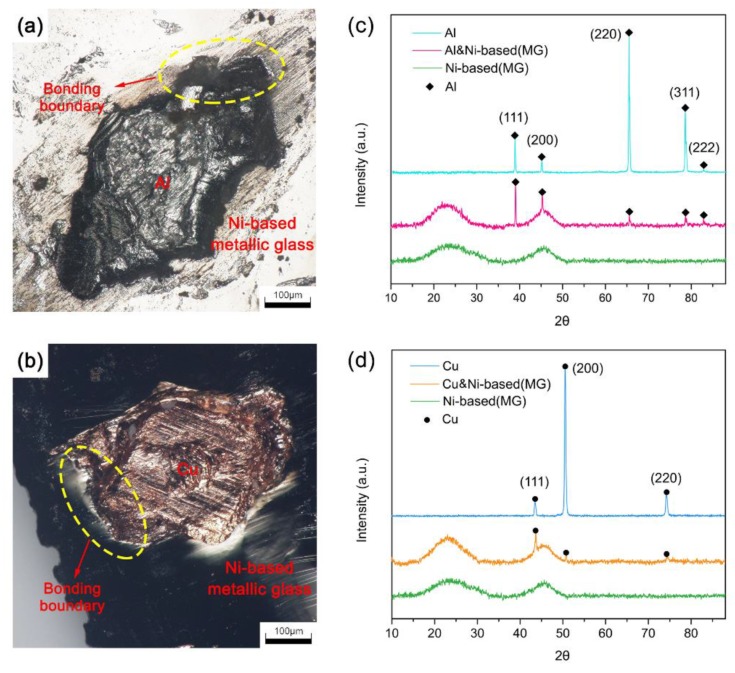
The joint surface morphology (**a**) of the Al/Ni-based (MG) composite sample at a bonding time of 80 ms, and the XRD results (**c**) of the joint surface and relative raw materials. The joint surface morphology (**b**) of the Cu/Ni-based (MG) composite sample at a bonding time of 60 ms, and the XRD results (**d**) of the joint surface and relative raw materials.

Figure 4a,b show a topographical view of the junction of the two materials after fracturing the consolidated samples under a digital microscope. The joint boundary shows that under ultrasonic vibrations, the metallic glass and crystalline metals can be bonded well in a solid state. Because the metallic glass has higher strength than the crystalline metals, the crystalline metals remained on the metallic glass ribbons after fracturing. The phases in the metallic glass, crystalline metals, and bonding interfaces were also analyzed by XRD. As shown in Figure 4c, the XRD pattern of the Ni-based metallic glass showed only two diffuse peaks. In contrast, Al showed five distinct, sharp diffraction peaks corresponding to its (111), (200), (220), (311), and (222) planes. The diffraction peaks at the joint interfaces of the Al/Ni-based (MG) composite samples were the superposition of the peaks for the Ni-based metallic glass and the Al, and no other crystalline peaks appeared, indicating no new substances formed at the joint interfaces. As shown in Figure 4d, the XRD pattern of the crystalline Cu had three distinct, sharp peaks corresponding to the (111), (200), and (220) planes. The diffraction peaks at the joint interfaces of the Cu/Ni-based (MG) composite samples were the superposition of the Ni-based metallic glass and the Cu diffraction peaks, and no other crystalline peaks appeared, indicating that no new substances formed at the joint interfaces and the metallic glass did not crystallize.

**Figure 5:**
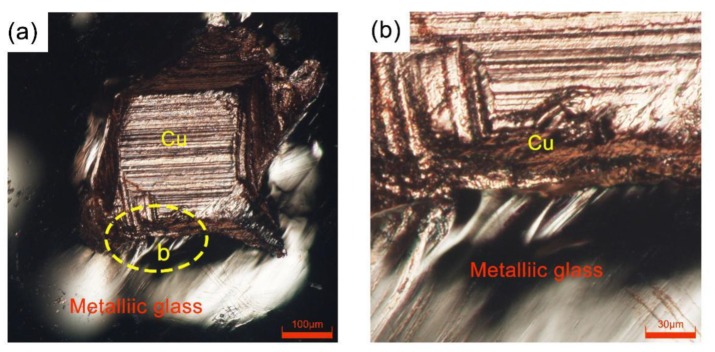
The joint surface morphology of the Cu/Ni-based (MG) composite sample at a bonding time of 140 ms (**a**), and a larger view of the boundary (**b**).

Table 2 shows the values of the force to tear the composite samples apart. When the bonding times were relatively short, the moderate input energy from the ultrasonic bonding system into the composite samples made the raw materials bond better, and the samples required a larger force to tear them apart. However, when the input energy was relatively excessive, the bonded samples required a smaller force to tear the raw materials apart. Figure 5a,b show the topographical view of the junction of the Cu/Ni-based (MG) composite samples after tearing apart at a bonding time of 140 ms. The crystalline metal remained on the metallic glass ribbon, and the fracturing boundary was relatively neat. This may be connected with the plastic flow of the raw materials during bonding, the excessive input energy resulting in more plastic flow of raw materials, and the area affected by ultrasonic vibrations being clearer. Then, the two materials were easier to be torn apart along the boundary of the ultrasonic-vibration-affected zone.

### 3.3. Hardness and Modulus Inside the Consolidated Samples

**Figure 6:**
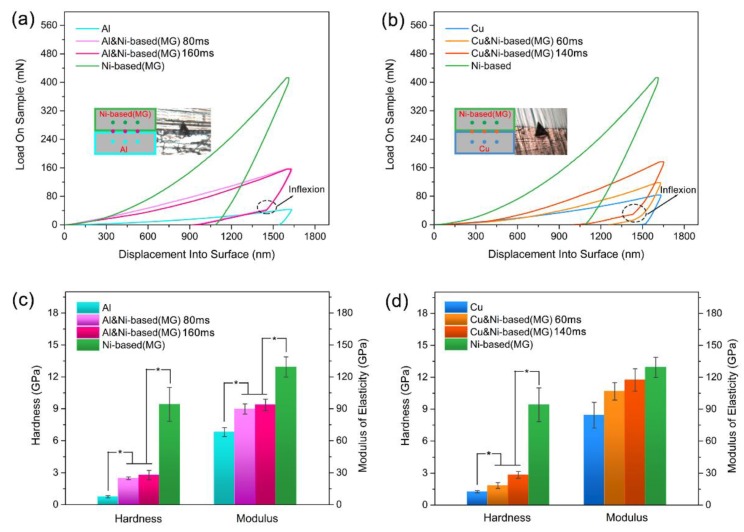
Nanoindentation curves (**a**) and the hardness and modulus (**c**) of the cross section of Al/Ni-based (MG) composite specimens at bonding times of 80 and 160 ms. Nanoindentation curves (**b**) and the hardness and modulus (**d**) of the cross section of the Cu/Ni-based (MG) composite samples at bonding times of 60 and 140 ms. Data presented as mean ± standard deviation, * *p* < 0.05.

To analyze how the ultrasonic vibration energy affected the mechanical properties of the consolidated metallic glass and crystalline metal composites, we tested the hardness and modulus of the Ni-based metallic glass, the crystalline metals, and consolidated specimens by nanoindentation. As shown in Figure 6a,b, during the nanoindentation test, three sampling points were selected in the middle of the raw materials’ cross sections and the joint interfaces of the composite samples, respectively. The depths of indentations were all 1600 nm to determine the critical compressive loads. The indentation curves in Figure 6a,b correspond to the hardness and modulus of the sampling points on the different sections selected closest to the average value of the section. The indentation curves of the consolidated samples were located between the indentation curves of the metallic glass and the crystalline metals. The compressive loads that the composite specimens could withstand were between the limited loads of the two materials. Also, the unloading curves of the consolidated samples had significant inflexion, mainly because the crystalline metals have higher plasticity than the metallic glass. For the same displacement into the surface, the indenter produced more permanent plastic deformation in the metallic glass than in the crystalline metals; thus, when the consolidated sample was unloaded, the indenter detached from the metallic glass before the crystalline metals. This may also be connected with the phase transformations and residual deformation of the raw materials [35,36]. In the process of loading, it was easier for the crystalline metal than the metallic glass to form a phase transition, and the residual deformation of the crystalline metal was smaller than that of the metallic glass after unloading.

Figure 6c,d show the hardness (H) and elastic modulus (E) of the cross sections of the Ni-based metallic glass, crystalline metals, and consolidated samples, respectively. The hardness and elastic modulus of the consolidated specimens were between the respective values of the two materials. The hardness values of Al, Cu, and Ni-based metallic glass were 0.75 ± 0.11, 1.26 ± 0.11, and 9.41 ± 1.59 GPa, respectively. Further, the hardness values of Al/Ni-based (MG) composites for bonding times of 80 and 160 ms as well as Cu/Ni-based (MG) composites for bonding times of 60 and 140 ms were 2.49 ± 0.13, 2.79 ± 0.44, 1.84 ± 0.27, and 2.84 ± 0.33 GPa, respectively. The hardness of the ultrasonically bonded samples was significantly different from that of the Ni-based metallic glass and crystalline metal materials, as revealed by Tukey’s test (*p* < 0.05). The modulus values of Al, Cu, and Ni-based metallic glass were 67.97 ± 4.20, 84.15 ± 12.07, and 129.16 ± 9.52 GPa, respectively. Further, the modulus values of Al/Ni-based (MG) composites for bonding times of 80 and 160 ms as well as Cu/Ni-based (MG) composites for bonding times of 60 and 140 ms were 89.68 ± 4.82, 93.53 ± 5.35, 106.58 ± 8.19, and 117.35 ± 10.47 GPa, respectively. The elastic modulus values of the Ni-based metallic glass and Al consolidated samples were significantly different from that of the two materials, as revealed by Tukey’s test (*p* < 0.05), but the elastic modulus values of the Ni-based metallic glass and Cu consolidated samples were not obviously different from that of the two materials. This result mainly occurred because of the slight difference in elastic modulus between Cu and the Ni-based metallic glass. As shown in Figure 3f, when the ultrasonic bonding system inputted too much energy into the interior of the consolidated sample, this caused penetration, which increased the error in the elastic modulus at the consolidation interface of the Cu/Ni-based (MG) composite samples relative to the Al/Ni-based (MG) composite samples. As the ultrasonic vibration energy inputted into the consolidated sample increased, the elastic modulus and hardness at the consolidation interfaces increased. This result may be connected with the plastic flow of the raw materials at the interface junction during bonding. The higher inputted ultrasonic energy may have caused the two materials to diffuse more deeply, which slightly increased the hardness and modulus. Table 3 shows the deformation relative to yielding (H/E) and the resistance to plastic indentation ratios (H^3^/E^2^) calculated based on nanoindentation results. H/E and H^3^/E^2^ ratios are important and valuable parameters for predicting the resistance of samples to plastic deformation [35,37]. A higher ratio means better sample resistance to plastic deformation. Table 3 illustrates that ultrasonic bonding additive manufacturing can combine the durability of metallic glass and crystalline metals.

## 4. Conclusions

Al/Ni-based (MG) and Cu/Ni-based (MG) composites were manufactured additively via ultrasonic vibrations. The range of the inputted ultrasonic vibration energy to bond Ni-based metallic glass and Al well was wider than that of Ni-based metallic glass and Cu. No intermetallic compounds formed at the junction of the metallic glass composite samples, and the Ni-based metallic glass did not crystallize after formation. The hardness and modulus of the interior of the composite specimens produced by ultrasonic additive manufacturing were between the respective values of the two materials. The mechanical properties of the metallic glass and crystalline metals were fused by ultrasonic vibrations. Ultrasonic bonding can also be combined with traditional machining or laser cutting to perform layer-by-layer cumulative formation. We believe that this technique will allow 3D printing of bulk, complex, high-performance structures of metallic glass composite parts and promote the development and application of metallic glass in industrial fields and others.

## Figures and Tables

**Table 1:** Ultrasonic bonding experiment parameters.

Material	Fixed Factors			Control Factors		
	Factor	Level	Unit	Factor	Value	Unit
Ni-based (MG) and Al	Pressure	0.18	MPa	Bonding time	60	ms
	Delay time	40	ms		80	
	Hold time	50	ms		160	
Ni-based (MG) and Cu	Pressure	0.18	MPa	Bonding time	40	ms
	Delay time	40	ms		60	
	Hold time	50	ms		140	

**Table 2:** The force to tear the samples apart.

Materials	Ni-based (MG) and Al			Ni-based (MG) and Cu		
Bonding time (ms)	60	80	160	40	60	140
Force (N)	11.71 ± 3.24	12.61 ± 1.22	7.23 ± 1.97	10.82 ± 0.90	10.71 ± 1.61	5.85 ± 1.50

**Table 3:** The deformation relative to yielding (H/E) and resistance to the plastic indentation (H^3^/E^2^).

Samples	H/E	H^3^/E^2^ (GPa)
Al	0.01111 ± 0.00185	0.00010 ± 0.00004
Ni-based (MG) and Al (80 ms)	0.02776 ± 0.00005	0.00192 ± 0.00009
Ni-based (MG) and Al (160 ms)	0.02977 ± 0.00368	0.00256 ± 0.00106
Ni-based (MG)	0.06726 ± 0.00279	0.03948 ± 0.00588
Ni-based (MG) and Cu (60 ms)	0.01722 ± 0.00125	0.00056 ± 0.00016
Ni-based (MG) and Cu (140 ms)	0.02413 ± 0.00099	0.00166 ± 0.00030
Cu	0.01527 ± 0.00356	0.00031 ± 0.00018
